# Lipoprotein(a) is a new prognostic factor in patients with psoriasis and coronary artery disease: a retrospective cohort study

**DOI:** 10.1186/s12944-023-01901-4

**Published:** 2023-09-02

**Authors:** Lin Zhao, Lin Sun, ZengLei Zhang, KunQi Yang, ZuoZhi Li, Man Wang, Yan Zeng, XianLiang Zhou, WeiXian Yang

**Affiliations:** Department of Cardiology, Fuwai Hospital, National Center for Cardiovascular Disease, Chinese Academy of Medical Sciences and Peking Union Medical College, No. 167, Beilishi Road, Beijing, 100037 China

**Keywords:** Lipoprotein(a), Coronary artery disease, Psoriasis, Prognosis

## Abstract

**Background:**

The prognostic value of lipoprotein (Lp) (a) in patients who have suffered from coronary artery disease (CAD) has not been fully studied, and the results are inconsistent. This study was conducted to evaluate whether increased Lp(a) concentrations cause differences in clinical adverse outcomes in patients with psoriasis who have already suffered from CAD.

**Methods:**

This retrospective cohort study included consecutive patients with psoriasis and CAD between January 2017 and May 2022 in our hospital. The clinical records were collected, and comparisons were made between patients in the low Lp(a) and high Lp(a) groups. Cox proportional hazard analysis and log-rank tests were used to evaluate the association between variables.

**Results:**

Among 295 patients, 148 patients were in the low Lp(a) group, and 147 were in the high Lp(a) group. These two groups did not differ significantly in age, gender or body mass index. Compared with the low Lp(a) group, the levels of platelet counts (*P* = 0.038) and high sensitivity C reactive protein (*P* = 0.012) were higher in the high Lp(a) group. Patients in the high Lp(a) group had higher total cholesterol levels (*P* = 0.029) and lower triglyceride levels (*P* = 0.037). Among the whole cohort, clinical adverse events were not correlated with Lp(a) concentrations after a median follow-up of 3 years. However, in the subgroup analysis, there were significant differences in all-cause death (log rank *P* = 0.036) and rehospitalization (log rank *P* = 0.037) between the two groups in patients with diabetes; a difference in rehospitalization (log rank *P* = 0.042) was also found between the two groups in men.

**Conclusions:**

In patients with psoriasis and CAD, high levels of Lp(a) were related to a poor prognosis, especially in patients with diabetes and in men. These results will provide valuable information for the risk stratification of patients with psoriasis and CAD.

**Supplementary Information:**

The online version contains supplementary material available at 10.1186/s12944-023-01901-4.

## Background

Lipoprotein(a) [Lp(a)] is a low-density lipoprotein (LDL)-like particle with an apolipoprotein (a) moiety bound to the apolipoprotein B component [1]. It is possible that Lp(a) contributes to cardiovascular disease via proatherogenic effects, proinflammatory effects, and prothrombotic effects of its LDL-like component, oxidized phospholipid and plasminogen-like apo(a), respectively [2]. Nevertheless, the prognostic value of Lp(a) in patients who have suffered from coronary artery disease (CAD) has not been fully studied, and the results are inconsistent. In patients with CAD, Lp(a) is positively correlated with adverse events in some studies [3–6]; in contrast, other studies have not found an increase in major adverse clinical outcomes among CAD patients with high Lp(a) concentrations [7, 8].

Psoriasis is a chronic inflammatory multisystemic skin condition that is linked with a number of comorbidities, affecting 2% of the global population [9]. It is estimated that more than half of patients present within the first three decades of their lives [10]. Evidence from clinical studies indicates that patients with psoriasis have an increased risk of cardiovascular disease [11, 12]. In comparison to control groups, people with psoriasis have a nearly six-year shorter life expectancy, and most deaths related to psoriasis are associated with cardiovascular morbidities [13]. To date, it is not clear whether increased Lp(a) concentrations cause differences in clinical adverse outcomes in patients with psoriasis who have already suffered from CAD. Thus, this study was conducted to evaluate this issue. These results will provide valuable information for risk stratification among these patients.

## Methods

### Study population

**Fig. 1:**
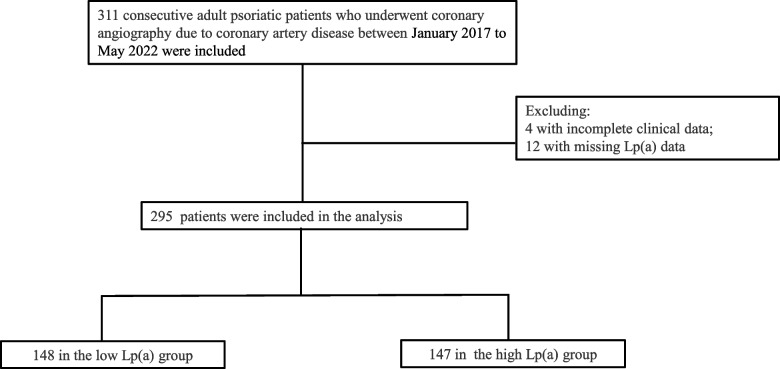
Flow chart showing the selection of patients

Three hundred and eleven adult patients with psoriasis who underwent coronary angiography for CAD at Fuwai Hospital, Beijing, China, between January 2017 and May 2022 were consecutively enrolled. Four patients with incomplete clinical information and twelve patients whose Lp(a) data were missing were excluded. Ultimately, 295 patients were included in this study. The flow chart of the included patients is shown in Fig. 1. Treatments included medication therapy alone, percutaneous coronary intervention, and coronary artery bypass grafting, all of which were in accordance with current guidelines and the patients’ preferences [14, 15]. This study was performed in accordance with the Declaration of Helsinki and approved by Fuwai Hospital’s Institute Ethics Committee. Data were anonymized and deidentified before being analyzed.

### Biochemical analysis and definitions

We collected the clinical data from medical records, including demographic data, laboratory measurements, pharmacological treatments and coronary angiography reports of the patients. A minimum of 12 h of fasting was required for all patients before venous blood was collected. The biochemical measurements were performed at Fuwai Hospital’s clinical chemistry department. An automatic biochemistry analyzer (Hitachi 7150, Tokyo, Japan) was used to measure the levels of serum triglycerides, high-density lipoprotein cholesterol, total cholesterol (TC) and LDL cholesterol (LDL-c). Immunoturbidimetry (Beckmann Assay 360, Bera, Calif., USA) was used to determine the levels of high sensitivity C-reactive protein (hs-CRP). Lp(a) levels were measured by immunoturbidimetry [LASAY Lp(a) auto; SHIMA laboratories; Tokyo, Japan]. According to whether their Lp(a) levels were higher or lower than the median level (14.4 mg/dL) of the whole cohort, patients were divided into two groups.

Diabetes mellitus was diagnosed when patients met one of the following criteria:asymptomatic patients with fasting plasma glucose ≥ 7.0 mmol/l, or with 2-h plasma glucose ≥ 11.1 mmol/l in 75g oral glucose tolerance tests; or patients with typical symptoms of diabetes combined with random blood glucose levels ≥11.1 mmol/l; or patients with an existing diagnosis of diabetes [16]. Hypertension was diagnosed according to the guideline [17]. The estimated glomerular filtration rate (eGFR) was calculated using the Chronic Kidney Disease Epidemiology Collaboration equation [18], and patients with an eGFR < 60 mL/min/1.73 m^2^ were diagnosed with chronic kidney disease (CKD). A nonbiologic systemic treatment for psoriasis included steroids and methotrexate, while biologics included interleukin 12/23, tumor necrosis factor alpha and interleukin 17 inhibitors. A minimum of six months of follow-up was provided to all patients after discharge. They were evaluated for the occurrence of all-cause death and rehospitalization due to heart failure or severe arrhythmias. A team of independent clinical physicians carefully reviewed and verified all events.

### Statistical analyses

Continuous variables were assessed for normality using the histogram and normal quantile–quantile plot. Continuous values were expressed as the mean ± standard deviation when they were normally distributed and were expressed as the median (25^th^, 75^th^ percentile) when they were not normally distributed. Continuous values were compared using Student’s t tests when they were normally distributed or using rank-sum tests when they were nonnormally distributed. Categorical variables were presented as numbers (percentage), and differences were detected using Pearson’s chi-square test or Fisher’s exact test. The parameters with *p* < 0.1 in the univariable Cox proportional hazard analysis were included in the multivariable Cox proportional hazard analysis to identify independent risk factors. The difference of the cumulative incidence of clinical events between the two groups was compared using log-rank tests. Subgroup analysis was conducted according to age (≤ 60 and > 60 years), gender, diabetes, hypertension and acute coronary disease (ACS). GraphPad Prism 8.0 (GraphPad, San Diego, CA, USA) was used to generate the Kaplan‒Meier curve. SPSS 25.0 (SPSS Inc., Chicago, Illinois, USA) was used to conduct all statistical analyses. The significance level was defined as a two-sided *P* value of less than 0.05.

## Results

### Clinical characteristics of the patients

Among 295 patients, 148 patients were in the low Lp(a) group, and 147 patients were in the high Lp(a) group. The baseline clinical characteristics of the overall population are detailed in Table 1. In total, 165 (55.9%) patients had hypertension, 122 (41.4%) had diabetes, and 278 (94.2%) had hyperlipidemia. Twenty-six (8.8%) patients had CKD. According to the results of the Psoriasis Area Severity Index score, the cohort had mild to moderate skin disease severity (median 6.2), and the mean psoriasis disease duration was 20 years. For the treatment of psoriasis, a total of 12.9% of patients used phototherapy, 41.0% used nonbiologic systemic treatment and 10.5% used biologic treatment. A total of 92.5% of patients were discharged to take aspirin, 81.2% to take P2Y12 inhibitors, 84.3% to take β-blockers, 50.5% to take angiotensin-converting enzyme inhibitors or angiotensin receptor blockers, 19.8% to take calcium channel blockers and 96.9% to take statins.

**Table 1:** Baseline clinical characteristics of patients

Parameter	Entire cohort (*n* = 295)	Low Lp(a) group (*n* = 148)	High Lp(a) group (*n* = 147)	*P*
Age, years (*n* = 295)	58.69 ± 9.82	58.39 ± 10.47	58.99 ± 9.14	0.604
Male, % (*n* = 295)	260(88.1)	133(89.9)	127(86.4)	0.357
BMI, kg/m^2^ (*n* = 295)	26.09 ± 3.33	26.47 ± 3.28	25.70 ± 3.35	0.097
Current smoker, % (*n* = 295)	195(66.1)	97(65.5)	98(66.7)	0.838
Hypertension, % (*n* = 295)	165(55.9)	87(58.8)	78(53.1)	0.322
Diabetes, % (*n* = 295)	122(41.4)	62(41.9)	60(40.8)	0.851
Hyperlipidemia, % (*n* = 295)	278(94.2)	140(94.6)	138(93.9)	0.792
Family history of CAD, % (*n* = 295)	41(13.9)	21(14.2)	20(13.6)	0.885
Previous stroke, % (*n* = 295)	27(9.2)	16(10.8)	11(7.5)	0.322
Peripheral vascular disease, % (*n* = 295)	15(5.1)	10(6.8)	5(3.4)	0.190
CKD, % (*n* = 295)	26(8.8)	10(6.8)	16(10.9)	0.211
ACS, % (*n* = 295)	186(63.1)	93(62.8)	93(63.3)	0.939
Psoriasis characteristics				
Psoriatic arthritis, % (*n* = 256)	10(3.9)	7(5.3)	3(2.4)	0.372
Disease duration, years (*n* = 267)	20(15, 30)	20(15, 30)	22.5(15, 30)	0.814
PASI score (*n* = 245)	6.2(2.2, 13.25)	5.5(1.1, 12.55)	7.9(3.8, 13.5)	0.500
Topical treatment, % (*n* = 256)	167(65.2)	88(67.2)	79(63.2)	0.504
Phototherapy, % (*n* = 256)	33(12.9)	18(13.7)	15(12.0)	0.678
Nonbiologic systemic treatment, % (*n* = 256)	105(41.0)	53(40.5)	52(41.6)	0.853
Biologic treatment, % (*n* = 256)	27(10.5)	12(9.2)	15(12)	0.460
Medication at discharge (*n* = 293)				
Aspirin, %	271(92.5)	138(93.2)	133(91.7)	0.622
P2Y12 inhibitors, %	238(81.2)	119(80.4)	119(82.1)	0.715
ACEIs/ARBs, %	148(50.5)	75(50.7)	73(50.3)	0.955
β-blockers, %	247(84.3)	117(79.1)	130(89.7)	0.013
Statin, %	284(96.9)	143(96.6)	141(97.2)	1.000
Calcium channel blockers, %	58(19.8)	33(22.3)	25(17.2)	0.278

*Abbreviations: ACS* Acute coronary syndrome, *ACEIs* Angiotensin-converting enzyme inhibitors, *ARBs* Angiotensin receptor blockers, *BMI* Body mass index, *CAD* Coronary artery disease, *CKD* Chronic kidney disease, *Lp(a)* Lipoprotein(a), *PASI* Psoriasis area severity index

The two groups did not differ significantly in age (*P* = 0.604), gender (*P* = 0.357), or body mass index (*P* = 0.097). The proportions of hypertension (*P* = 0.322), diabetes (*P* = 0.851), hyperlipidemia (*P* = 0.792) and CKD (*P* = 0.211) were not significantly different between the low Lp(a) group and high Lp(a) group (Table 1). There were no significant differences between the groups in the Psoriasis Area Severity Index scores (*P* = 0.500) or disease duration of psoriasis (*P* = 0.814). Regarding the biochemical and coronary characteristics (Table 2), the platelet counts (*P* = 0.038) and hsCRP levels (*P* = 0.012) were higher in the high Lp(a) group than in the low Lp(a) group. Despite having similar levels of LDL-c (*P* = 0.174) and high-density lipoprotein cholesterol (*P* = 0.168), patients in the high Lp(a) group had higher TC levels (*P* = 0.029) and lower triglyceride levels (*P* = 0.037). Right coronary artery involvement was more prone to occur in the high Lp(a) group (78.1% vs. 67.3%, *P* = 0.039), while the tendency of left anterior descending artery (*P* = 0.296) and left circumflex artery involvement (*P* = 0.968) and the number of vessels involved were not significantly different between the groups. We used the Framingham risk score to evaluate the 10-year cardiovascular disease risk in these patients, and there were no significant differences between the two groups (Table 2).

**Table 2:** Biochemical results, coronary characteristics and clinical outcomes of patients

Parameter	Entire cohort (*n* = 295)	Low Lp(a) group (*n* = 148)	High Lp(a) group (*n* = 147)	*P*
Laboratory values				
Platelet count, × 10^9^/L (*n* = 295)	224(184, 262)	216(180, 257)	233(187, 275)	0.038
hsCRP, mg/L (*n* = 272)	1.57(0.65, 3.72)	1.34(0.62, 2.99)	1.85(0.70, 5.76)	0.012
eGFR, ml/min/1.73m^2^ (*n* = 295)	87.50 ± 20.03	89.09 ± 19.87	85.91 ± 20.13	0.174
TC, mmol/L (*n* = 295)	3.82(3.22, 4.46)	3.71(3.13, 4.40)	3.88(3.42, 4.56)	0.029
LDL-c, mmol/L (*n* = 295)	2.26(1.76, 2.86)	2.18(1.61, 2.94)	2.29(1.86, 2.82)	0.174
HDL-c, mmol/L (*n* = 295)	1.06(0.90, 1.26)	1.04(0.88, 1.22)	1.11(0.92, 1.29)	0.168
Triglycerides, mmol/L (*n* = 295)	1.49(1.08, 2.00)	1.53(1.10, 2.20)	1.40(1.06, 1.83)	0.037
Coronary characteristics				
Presence of plaque, % (*n* = 293)	289(98.6)	144(98)	145(99.3)	0.619
Culprit vessel (*n* = 293)				
LAD, %	253(86.3)	130(88.4)	123(84.2)	0.296
LCX, %	199(67.9)	100(68.0)	99(67.8)	0.968
RCA, %	213(72.7)	99(67.3)	114(78.1)	0.039
LM, %	45(15.4)	27(18.4)	18(12.3)	0.152
No. of diseased vessels (*n* = 293)				
1, %	65(22.2)	33(22.4)	32(21.9)	0.913
2, %	72(24.6)	37(25.2)	35(24.0)	0.812
3, %	152(51.9)	74(50.3)	78(53.4)	0.597
Target lesion morphology				
Bifurcation lesion, % (*n* = 175)	22(12.6)	8(9.5)	14(15.4)	0.243
Chronic total occlusion, % (*n* = 175)	46(26.3)	20(23.8)	26(28.6)	0.475
Complex lesion (lesion of type B2 or C), % (*n* = 175)	140(80)	63(75)	77(84.6)	0.112
PCI, % (*n* = 295)	172(58.3)	82(55.4)	90(61.2)	0.311
Framingham risk score for estimation of 10-years of cardiovascular diseases risk				
Low-risk, % (*n* = 295)	46(15.6)	23(15.5)	23(15.6)	0.980
Moderate-risk, % (*n* = 295)	28(9.5)	13(8.8)	15(10.2)	0.677
High-risk, % (*n* = 295)	221(74.9)	112(75.7)	109(74.1)	0.762
CABG, % (*n* = 295)	22(7.5)	12(8.1)	10(6.8)	0.670
Follow-up time, months (*n* = 280)	36(20, 52)	35(21.75, 50.25)	37(18.75, 53)	0.847
All-cause death, % (*n* = 280)	10(3.6)	3(2.1)	7(5.1)	0.311
Rehospitalization, % (*n* = 280)	12(4.3)	3(2.1)	9(6.5)	0.069

*Abbreviations: CABG* Coronary artery bypass grafting, *eGFR* Estimated glomerular filtration rate, *HDL-c* High-density lipoprotein cholesterol, *hsCRP* High-sensitivity C-reactive protein, *LAD* Left anterior descending artery, *LCX* Left circumflex artery, *LDL-c* Low-density lipoprotein cholesterol, *LM* Left main artery, *Lp(a)* Lipoprotein(a), *PCI* Percutaneous coronary intervention, *RCA* Right circumflex artery, *TC* Total cholesterol

### Clinical outcomes of the patients

**Fig. 2:**
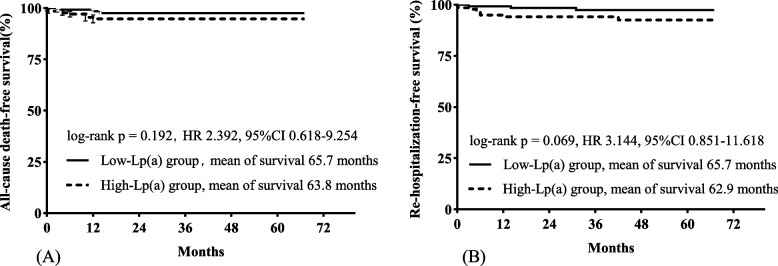
The results of log-rank tests (Kaplan‒Meier survival curves) estimated clinical outcomes for patients in the low and high Lp(a) groups. **A** Survival curves for all-cause death; **B** survival curves for rehospitalization. Lp(a), lipoprotein(a)

**Fig. 3:**
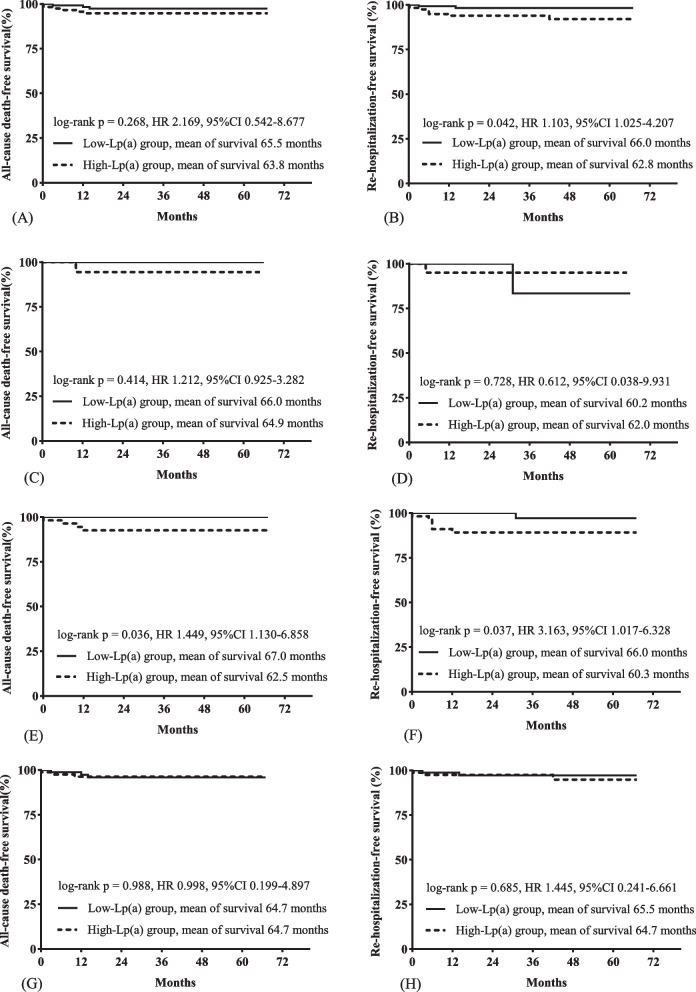
The results of log-rank tests (Kaplan‒Meier survival curves) estimated clinical outcomes for patients in the subgroups. **A** Survival curves for all-cause death in men; **B** survival curves for rehospitalization in men; **C** survival curves for all-cause death in women; **D** survival curves for rehospitalization in women; **E** survival curves for all-cause death in patients with diabetes; **F** survival curves for rehospitalization in patients with diabetes; **G** survival curves for all-cause death in patients without diabetes; **H** survival curves for rehospitalization in patients without diabetes. Lp(a), lipoprotein(a)

A total of 280 patients were followed up for a median of 36 months, including 142 in the low Lp(a) group and 138 in the high Lp(a) group. There were no significant differences between the two groups in all-cause death (*P* = 0.311) or rehospitalization rates (*P* = 0.069) (Table 2). The results of log-rank tests found the same trends [for all-cause death, log-rank *P* = 0.192 (mean of survival in the low Lp(a) group, 65.7 months; mean of survival in the high Lp(a) group, 63.8 months; for rehospitalization, log-rank *P* = 0.069 (mean of survival in the low Lp(a) group, 65.7 months; mean of survival in the high Lp(a) group, 62.9 months] (Fig. 2). Similarly, the results of univariable Cox proportional hazard analysis showed that high Lp(a) was not associated with all-cause death [HR (hazard ratio) 2.392, 95% CI (95% confidence interval) 0.618–9.254, *P* = 0.206) or rehospitalization (HR 3.144, 95% CI 0.851–11.618, *P* = 0.086) in all patients (Table 3). Significant differences were found in the results of the subgroup analysis. Among men, the risk of rehospitalization was higher in the high Lp(a) group than in the low Lp(a) group (log rank *P* = 0.042; mean of survival in the low Lp(a) group, 66.0 months; mean of survival in the high Lp(a) group, 62.8 months). In patients with diabetes, those in the high Lp(a) group also had a higher risk of all-cause death (log rank *P* = 0.036; mean of survival in the low Lp(a) group, 67.0 months; mean of survival in the high Lp(a) group, 62.5 months) and rehospitalization (log rank *P* = 0.037; mean of survival in the low Lp(a) group, 66.0 months; mean of survival in the high Lp(a) group, 60.3 months) than those in the low Lp(a) group (Fig. 3). The results of univariable Cox proportional hazard analysis also showed that high Lp(a) was associated with all-cause death (HR 1.449, 95% CI 1.130–6.858, *P* = 0.033) and rehospitalization (HR 3.163, 95% CI 1.017–6.328, *P* = 0.045) in patients with diabetes; high Lp(a) was associated with rehospitalization in men (HR 1.103, 95% CI 1.025–4.207, *P* = 0.043) (Table 3). These differences were still significant (*P* < 0.05) after adjustment (Tables 4, 5 and 6).

**Table 3:** The results of univariable analysis

	**COX proportional hazard analysis**			**Log-rank tests**
	**HR**	**95%CI**	*P*	*P*
All-cause death				
All cohort	2.392	0.618–9.254	0.206	0.192
Subgroups				
Men	2.169	0.542–8.677	0.274	0.268
Women	1.212	0.925–3.282	0.515	0.414
Patients with DM	1.449	1.130–6.858	0.033	0.036
Patients without DM	0.988	0.199–4.897	0.988	0.988
Patients with age < 60y	1.347	0.311–6.020	0.697	0.695
Patients with age ≥ 60y	1.452	0.998–7.004	0.701	0.080
Patients with hypertension	1.975	0.472–8.266	0.352	0.342
Patients without hypertension	1.354	0.992–6.872	0.188	0.197
Patients with ACS	1.980	0.363–10.817	0.430	0.421
Patients without ACS	3.221	0.335–11.966	0.311	0.284
Rehospitalization				
All cohort	3.144	0.851–11.618	0.086	0.069
Subgroups				
Men	1.103	1.025–4.207	0.043	0.042
Women	0.612	0.038–9.931	0.730	0.728
Patients with DM	3.163	1.017–6.328	0.045	0.037
Patients without diabetes	1.445	0.241–6.661	0.687	0.685
Patients with age < 60y	2.545	0.494–8.120	0.264	0.247
Patients with age ≥ 60y	4.474	0.499–9.092	0.181	0.142
Patients with hypertension	2.022	0.483–8.064	0.335	0.325
Patients without hypertension	1.011	0.928–7.003	0.619	0.066
Patients with ACS	2.997	0.447–9.761	0.215	0.180
Patients without ACS	2.828	0.548–11.599	0.214	0.192

HRs and 95% CIs were calculated for the high lipoprotein (a) group relative to the low lipoprotein (a) group

*Abbreviations: ACS* Acute coronary syndrome, *DM* Diabetes, *HR* Hazard ratio, *95% CI* 95% Confidence interval

**Table 4:** The results of Cox proportional hazard analysis of risk factors for rehospitalization in men

Parameter	Univariable cox proportional hazard analysis			Multivariable cox proportional hazard analysis		
	HR	95%CI	*P*	HR	95%CI	*P*
Age	1.031	0.966–1.100	0.355	1.036	0.899–1.194	0.626
BMI	1.081	0.903–1.294	0.396	0.561	0.311–1.012	0.055
Diabetes	1.422	0.412–4.912	0.578			
Hypertension	1.277	0.360–4.526	0.705			
ACS	0.392	0.110–1.388	0.147			
PASI	1.103	1.008–1.207	0.032	1.099	0.991–1.219	0.075
Platelet count	1.001	0.991–1.011	0.876			
hsCRP	1.126	0.990–1.281	0.07	1.094	0.767–1.561	0.620
TC	0.775	0.402–1.494	0.446			
LDL-c	0.659	0.286–1.520	0.328			
HDL-c	0.725	0.104–5.059	0.745			
Triglycerides	0.529	0.179–1.563	0.250			
High Lp(a)	1.103	1.025–4.207	0.043	1.101	1.020–4.657	0.047

*Abbreviations: ACS* Acute coronary syndrome, *BMI* Body mass index, *HDL-c* High-density lipoprotein cholesterol, *HR* Hazard ratio, *hsCRP* High-sensitivity C-reactive protein, *LDL-c* Low-density lipoprotein cholesterol, *Lp(a)* Lipoprotein(a), *PASI* Psoriasis area severity index, *TC* Total cholesterol, *95% CI* 95% Confidence interval

**Table 5:** The results of Cox proportional hazard analysis of risk factors for all-cause death in patients with diabetes

Parameter	Univariable cox proportional hazard analysis			Multivariable cox proportional hazard analysis		
	HR	95%CI	*P*	HR	95%CI	*P*
Age	0.949	0.836–1.078	0.423	0.694	0.028–17.21	0.824
Male	1.014	0.962–1.069	0.597	1.021	0.927–1.093	0.662
BMI	0.936	0.694–1.262	0.665	0.243	0–137.719	0.662
Hypertension	1.827	0.191–17.672	0.599			
ACS	0.564	0.079–4.013	0.567			
PASI	1.003	1.000–1.005	0.069	1.005	0.942–1.071	0.886
Platelet count	1.008	0.997–1.019	0.142			
hsCRP	1.449	1.130–1.858	0.003	1.497	0.021–10.26	0.852
TC	1.306	0.554–3.075	0.542			
LDL-c	1.569	0.569–4.327	0.384			
HDL-c	0.259	0.006–12.028	0.490			
Triglycerides	0.183	0.019–1.758	0.141			
High Lp(a)	1.449	1.130–6.858	0.033	1.654	1.089–7.023	0.039

*Abbreviations: ACS* Acute coronary syndrome, *BMI* Body mass index, *HDL-c* High-density lipoprotein cholesterol, *HR* Hazard ratio, *hsCRP* High-sensitivity C-reactive protein, *LDL-c* Low-density lipoprotein cholesterol, *Lp(a)* Lipoprotein(a), *PASI* Psoriasis area severity index, *TC* Total cholesterol, *95% CI* 95% Confidence interval

**Table 6:** The results of Cox proportional hazard analysis of risk factors for rehospitalization in patients with diabetes

Parameter	Univariable cox proportional hazard analysis			Multivariable cox proportional hazard analysis		
	HR	95%CI	*P*	HR	95%CI	*P*
Age	1.022	0.930–1.124	0.652	0.999	0.822–1.214	0.991
Male	0.351	0.068–1.809	0.211	0.788	0.02–31.249	0.899
BMI	0.973	0.783–1.210	0.806	1.197	0.822–1.630	0.254
Hypertension	1.544	0.299–7.962	0.604			
ACS	0.216	0.042–1.116	0.067	0.075	0.002–2.692	0.156
PASI	1.053	0.930–1.191	0.415	1.001	0.996–1.004	0.936
Platelet count	1.008	1.000–1.016	0.057	1.007	0.988–1.027	0.456
hsCRP	1.163	1.017–1.328	0.027	1.295	0.947–1.771	0.105
TC	0.538	0.229–1.267	0.156			
LDL-c	0.376	0.109–1.300	0.122			
HDL-c	0.449	0.032–6.337	0.553			
Triglycerides	0.643	0.232–1.786	0.397			
High Lp(a)	3.163	1.017–6.328	0.045	3.127	1.012–6.598	0.048

*Abbreviations: ACS* Acute coronary syndrome, *BMI* Body mass index, *HDL-c* High-density lipoprotein cholesterol, *HR* Hazard ratio, *hsCRP* High-sensitivity C-reactive protein, *LDL-c* Low-density lipoprotein cholesterol, *Lp(a)* Lipoprotein(a), *PASI* Psoriasis area severity index, *TC* Total cholesterol, *95% CI* 95% Confidence interval

## Discussion

Psoriasis is a chronic inflammatory multisystemic skin condition that affects skin, and many other conditions could be associated with it, such as psoriatic arthritis, uveitis, depression, and inflammatory bowel diseases [10, 19]. Scaly skin patches, plaques, and pustules are common signs of the disease, along with episodes of remission and relapse. It has been reported that psoriasis is associated with an elevated prevalence of risk factors associated with cardiovascular diseases, such as obesity, diabetes, hyperlipidemia, and hypertension [20]. Clinical researchers have indicated that patients with psoriasis are more prone to develop cardiovascular disease [11, 12, 21–23]. Cardiovascular disorders and psoriasis are likely related owing to shared inflammatory factors influenced by genetic and molecular pathways between the two diseases [24]. Shared chronic inflammatory factors have various effects on the endothelium, leading to proatherogenic phenotype production [24]. Unfortunately, studies on the risk factors associated with clinical adverse events in patients who suffer from psoriasis and CAD are limited. To date, no studies have evaluated the effects of Lp(a) on the prognosis of patients with psoriasis and CAD. This research was performed to evaluate the effects of Lp(a) concentrations on clinical adverse events in these patients. We found that high Lp(a) levels were positively related to all-cause death and rehospitalization in patients with diabetes and in men. The risk assessment for patients with psoriasis and CAD will benefit from our results.

It is controversial whether Lp(a) concentrations and adverse clinical events are related in patients with CAD. In reported studies, Xu et al. [8] reported that there was no correlation between Lp(a) concentrations and cardiovascular outcomes during an average of 874 days of follow-up in Chinese patients who underwent percutaneous coronary intervention. In another study, in patients with acute coronary syndromes after coronary stenting, major cardiac events were not independently predicted by increased Lp(a) levels during a median 24-month follow-up [7]. According to Kardys et al. [25], in patients with complex disease, the level of Lp(a) was not associated with long-term prognosis (median 6 years), but 1-year major adverse cardiac events could be predicted by a high Lp(a) level. Other studies also reported a positive correlation between Lp(a) concentrations and cardiovascular events in patients with CAD [3, 4, 6, 26]. Xue et al. [3] found that after a median follow-up of 930 days in patients who underwent percutaneous coronary intervention due to ST-elevation myocardial infarction, atherosclerosis burden and mortality were related to Lp(a) levels. Liu et al. showed that [4] in patients with stable CAD, high Lp(a) levels may increase the risk of cardiovascular adverse events. Cui et al. [26] found that after 2.4 years of follow-up, an elevated Lp(a) level was significantly associated with a greater risk of major adverse cardiovascular and cerebrovascular events in CAD patients who underwent percutaneous coronary intervention. In our study, among the whole cohort, clinical adverse events did not correlate with Lp(a) concentrations after a median 3-year follow-up.

It is possible that Lp(a) levels may be responsible for the contradictory results in above studies. There is already evidence showing a correlation between high Lp(a) ≥ 30 mg/dl and increased risk of cardiovascular disease and all-cause death in patients with CAD [5, 27]. Another study [28] reported that those with Lp(a) ≥ 120 mg/dl had a 3- to 4-fold increase in myocardial infarction risk. However, in Gencer et al.’s study [29], cardiovascular outcomes were not predicted by high Lp(a) levels (≥ 30 mg/dl) in otherwise medically well-controlled patients. Several factors could contribute to conflicting results across these studies, including different inclusion criteria, study designs, follow-up times, and sample sizes. In addition, the levels of Lp(a) are affected by many factors, such as ethnic groups, different areas, combined disease, or the methods for measuring Lp(a) [30, 31]. Paré G. et al. [30] found that Lp(a) levels were lowest among Chinese patients (median 7.8 mg/dL) and highest among Africans (median 27.2 mg/dL). South Asians and Latin Americans carried an especially high population burden of higher Lp(a) levels. Waldeyer C. et al. [31] showed that in the European population, compared with the central (median 7.9 mg/dL) and southern European cohorts (median 10.9 mg/dL), northern European cohorts had lower Lp(a) levels (median 4.9 mg/dL). Therefore, it is possible for Lp(a) concentrations to fluctuate in different studies, which may affect the results of the studies. According to our data, the median level of Lp(a) was 14.4 mg/dl, which is relatively low and may explain the results that clinical adverse events did not correlate with Lp(a) levels in the whole cohort. Furthermore, most of the patients also underwent moderate- or high-intensity statin therapy, and in this study, the mean LDL-c level was 2.26 mmol/L; the association between Lp(a) and cardiovascular outcomes may be affected by this low level of LDL-c. Because of the inconsistent effects of Lp(a) levels on cardiovascular outcomes in patients with different clinical characteristics in above studies, the relationship between Lp(a) concentrations and cardiovascular events in patients with psoriasis and CAD needs to be explored in more studies.

Although an association between Lp(a) concentrations and clinical adverse events was not found in the whole cohort, significant associations were found in subgroups. The link between Lp(a) levels and clinical adverse events in patients with diabetes was also reported previously. According to Zhang et al. [32], increased Lp(a) levels were independently linked to both the presence and severity of CAD in patients with diabetes. Waldeyer C et al. [31] performed a meta-analysis of 7 cohorts, and the maximum follow-up was 24 years. They found that the increased risk of cardiovascular events was associated with a high level of Lp(a), particularly in patients with diabetes. The association between gender and Lp(a) concentrations in clinical outcomes was inconsistent. In Xu et al.’s study [33], the interaction between Lp(a) and gender showed a stronger association between Lp(a) and clinical adverse events in women than in men. However, in our study, the levels of Lp(a) were not significantly different between men and women; in the subgroup analysis, the association between high Lp(a) concentrations and clinical adverse events was found only in men and not in women. The following points should be noted. Although we included all patients with psoriasis and CAD in the last 5 years, the number of women in our cohort was still limited, which may affect the results of this study. The relationship between Lp(a) concentrations and sex in clinical adverse events needs to be explored further.

Multiple mechanisms are involved in the contribution of Lp(a) to CAD risk. Lp(a) promotes the expression of proinflammatory cytokines and induces endothelium activation; as a result, adhesion molecules are expressed, and inflammatory cells invade the arterial wall. Combining inflammation and Lp(a) may exacerbate endothelial dysfunction and loss of function, which further amplifies the loss of integrity and protective functions of the endothelium [34]. Additionally, the oxidized phospholipids in Lp(a) may contribute to facilitating fibrinolysis and have a pathophysiological role in atherothrombosis [2, 35].

The differences in clinical features and biochemical and coronary characteristics between patients in the high Lp(a) group and low Lp(a) group in our study are consistent with reported studies. Many studies have reported that patients have higher levels of hsCRP and total cholesterol [8, 26, 36], as well as higher levels of platelets [3], in the high Lp(a) level group. A previous study also reported [8] that the level of triglycerides was lower in the high Lp(a) group than in the low Lp(a) group. More patients in the high Lp(a) group had right coronary artery involvement in the present study, which was also found in Xu et al.’s study [8].

The effects of lowering Lp(a) levels on cardiovascular health have not been well confirmed by clinical trials, but there is a possible relationship between the treatment of lowering Lp(a) levels and cardiovascular benefits for patients with increased Lp(a) levels based on the possible association between Lp(a) concentrations and cardiovascular diseases. O'Donoghue et al. reported that a significant reduction in Lp(a) levels was observed in patients using evolocumab (a PCSK9 inhibitor); those with higher baseline Lp(a) levels had greater reductions and tended to benefit from PCSK9 inhibition [37]. The results of the ODYSSEY OUTCOMES trial reported that among patients with recent acute coronary syndromes, alirocumab independently reduced cardiovascular events by lowering Lp(a), and cardiovascular events were predicted to be reduced by 2.5% with a 5-mg/dl reduction in Lp(a) levels [38]. Another study found that in patients with elevated Lp(a) levels and established cardiovascular disease treated with APO(a)-L_Rx_, Lp(a) levels decreased dose-dependently [39]. More studies are needed to evaluate the impact of lowering Lp(a) levels on cardiac adverse events in patients.

### Study strengths and limitations

This is the first study to evaluate the effects of Lp(a) levels on clinical adverse outcomes in patients with psoriasis and CAD. We believe that our study makes a significant contribution to the literature because it shows that in patients with psoriasis and CAD, increased Lp(a) levels are linked with a poor prognosis in men and in patients with diabetes. These findings have important implications for the risk assessment of these patients. However, there were several limitations in this study. First, the study was conducted at a single center, which may lead to selection bias. A multicenter study with a larger sample size needs to be conducted to obtain more reliable conclusions. Second, in this study, Lp(a) was only measured at baseline, the levels of Lp(a) during the follow-up period may also be clinically significant. Last but not least, to better explain the impact of Lp(a) levels on long-term outcomes, the follow-up period needs to be extended.

## Conclusion

In patients with psoriasis and CAD, an increased Lp(a) level is linked with a poor prognosis in men and in patients with diabetes. The results suggest that Lp(a) may help the risk stratification of patients with psoriasis and CAD. These findings need more detailed studies to confirm, and it is also worth investigating whether lowering Lp(a) levels could improve the prognosis of patients with increased Lp(a) levels.

### Supplementary Information

**Additional file 1: Figure S1.** The results of log-rank tests (Kaplan‒Meier survival curves) estimated clinical outcomes in patients in subgroups. (A) survival curves for all-cause death in patients with age < 60 years old; (B) survival curves for rehospitalization in patients with age < 60 years old; (C) survival curves for all-cause death in patients with age ≥ 60 years old;(D) survival curves for rehospitalization in patients with age ≥ 60 years old; (E) survival curves for all-cause death in patients with hypertension; (F) survival curves for rehospitalization in patients with hypertension. Lp(a), lipoprotein(a). **Figure S2.** The results of log-rank tests (Kaplan‒Meier survival curves) estimated clinical outcomes in patients in subgroups. (A) Survival curves for all-cause death in patients without hypertension; (B) survival curves for rehospitalization in patients without hypertension; (C) survival curves for all-cause death in patients with ACS; (D) survival curves for rehospitalization in patients with ACS; (E) survival curves for all-cause death in patients without ACS; (F) survival curves for rehospitalization in patients without ACS. ACS, acute coronary disease; Lp(a), lipoprotein(a).

## Data Availability

The data used to support the findings of this study are available from the corresponding authors upon request.

## Abbreviations

ACS: Acute coronary disease
CAD: Coronary artery disease
CKD: Chronic kidney disease
eGFR: Estimated glomerular filtration rate
hsCRP: High sensitivity C-reactive protein
LDL: Low-density lipoprotein
LDL-c: LDL cholesterol
Lp(a): Lipoprotein(a)
TC: Total cholesterol
