# Supplementary material for: Lipoprotein(a) is a new prognostic factor in patients with psoriasis and coronary artery disease: a retrospective cohort study
Source: Lipids Health Dis. 2023 Sep 2;22:141. doi: 10.1186/s12944-023-01901-4 (PMC10474627; doi:10.1186/s12944-023-01901-4)
Supplement: Supplementary file 1 — Additional file 1: Figure S1. The results of log-rank tests (Kaplan‒Meier survival curves) estimated clinical outcomes in patients in subgroups. (A) survival curves for all-cause death in patients with age < 60 years old; (B) survival curves for rehospitalization in patients with age < 60 years old; (C) survival curves for all-cause death in patients with age ≥ 60 years old;(D) survival curves for rehospitalization in patients with age ≥ 60 years old; (E) survival curves for all-cause death in patients with hypertension; (F) survival curves for rehospitalization in patients with hypertension. Lp(a), lipoprotein(a). Figure S2. The results of log-rank tests (Kaplan‒Meier survival curves) estimated clinical outcomes in patients in subgroups. (A) Survival curves for all-cause death in patients without hypertension; (B) survival curves for rehospitalization in patients without hypertension; (C) survival curves for all-cause death in patients with ACS; (D) survival curves for rehospitalization in patients with ACS; (E) survival curves for all-cause death in patients without ACS; (F) survival curves for rehospitalization in patients without ACS. ACS, acute coronary disease; Lp(a), lipoprotein(a). [file 12944_2023_1901_MOESM1_ESM.pdf]

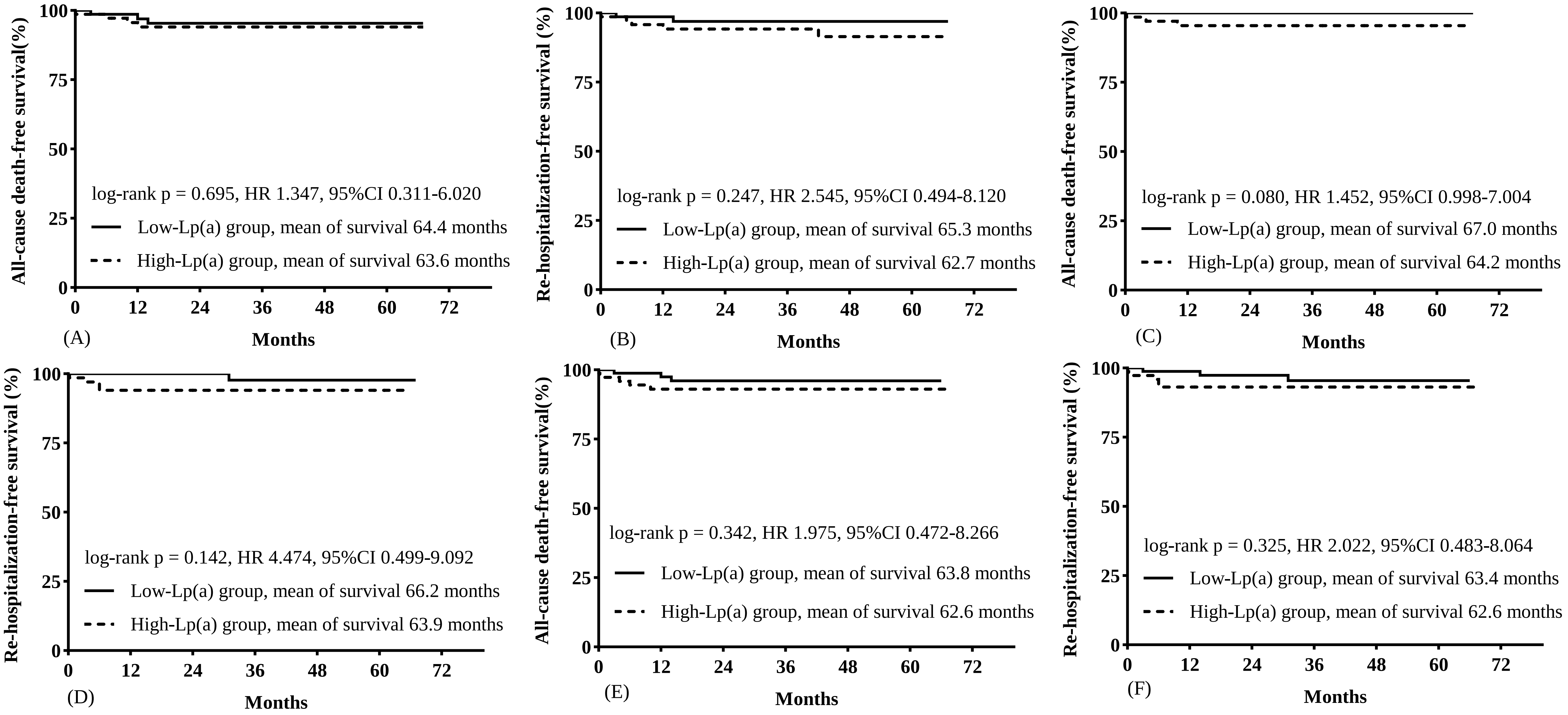

Figure S1. The results of log-rank tests (Kaplan–Meier survival curves) estimated clinical outcomes in patients in subgroups.(A) survival curves for all-cause death in patients with age < 60 years old; (B) survival curves for rehospitalization in patients with age < 60 years old; (C) survival curves for all-cause death in patients with age ≥ 60 years old;(D) survival curves for rehospitalization in patients with age ≥ 60 years old; (E) survival curves for all-cause death in patients with hypertension; (F) survival curves for rehospitalization in patients with hypertension. Lp(a), lipoprotein(a).

Lp(a), lipoprotein(a).

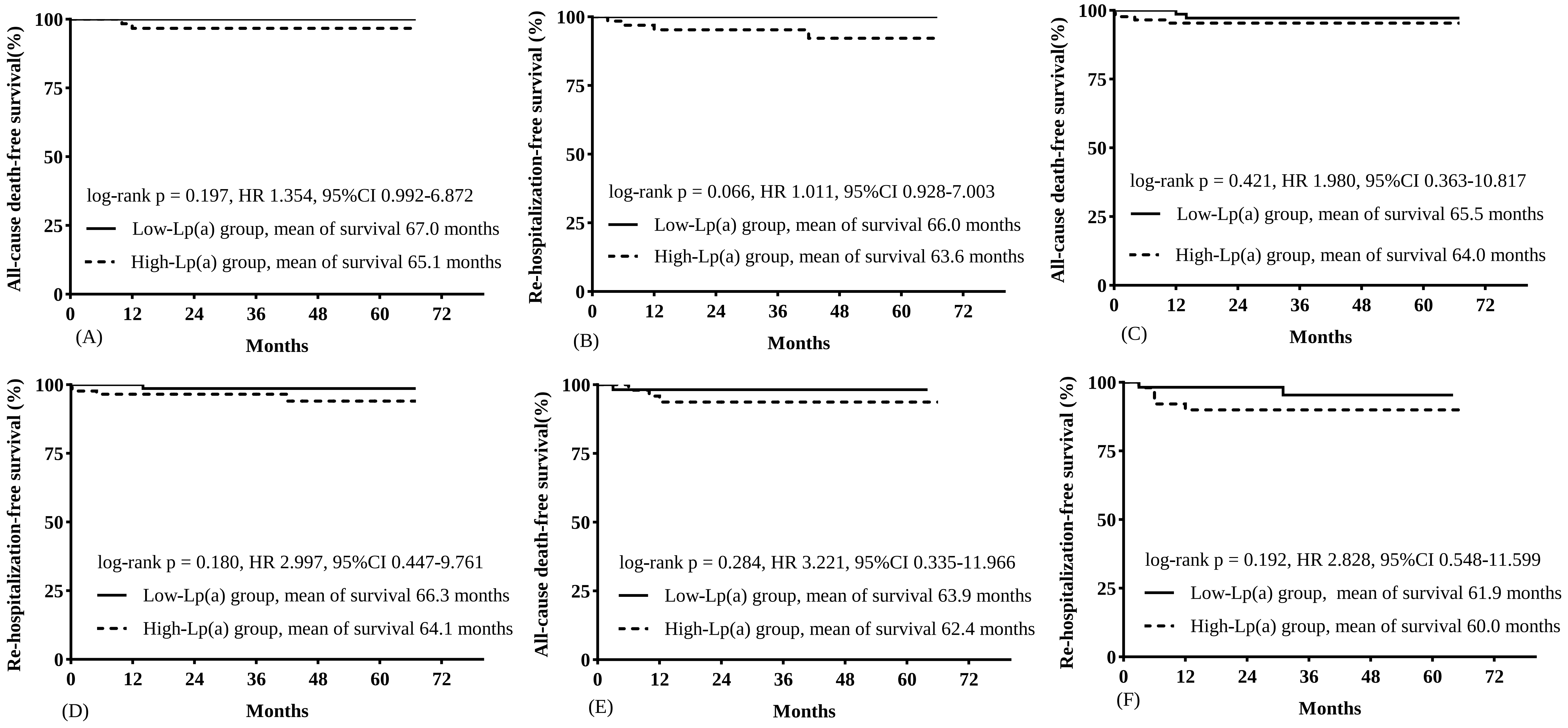

Figure S2. The results of log-rank tests (Kaplan–Meier survival curves) estimated clinical outcomes in patients in subgroups. (A) survival curves for all-cause death in patients without hypertension; (B)survival curves for re-hospitalization in patients without hypertension; (C) survival curves for all-cause death in patients with ACS; (D) survival curves for re-hospitalization in patients with ACS; (E) survival curves for all-cause death in patients without ACS; (F) survival curves for re-hospitalization in patients without ACS.

ACS, acute coronary disease; Lp(a), lipoprotein(a).
